# The *Black Voices in Research* curriculum to promote diversity and inclusive excellence in biomedical research

**DOI:** 10.1017/cts.2021.869

**Published:** 2021-10-14

**Authors:** Yulia A. Levites Strekalova, Yufan Sunny Qin, Shubam Sharma, Justine Nicholas, Gailine P. McCaslin, Kristina E. Forman, Denise B. Long, Tiffany Danielle Pineda, Taylor K. Williams, H. Robert Kolb

**Affiliations:** 1 Department of Health Services Research, College of Public Health and Health Professions, University of Florida, Gainesville, FL, USA; 2 Clinical and Translational Science Institute, University of Florida, Gainesville, FL, USA; 3 College of Journalism and Communications, University of Florida, Gainesville, FL, USA; 4 Department of Psychological Science, Radow College of Humanities and Social Sciences, Kennesaw State University, Kennesaw, GA, USA; 5 Department of Pediatrics, College of Medicine, University of Florida, Gainesville, FL, USA; 6 Division of Research Operations and Services, University of Florida, Gainesville, FL, USA; 7 Social and Behavioral Institutional Review Board Committee, University of Florida, Gainesville, FL, USA; 8 Innovation Academy, University of Florida, Gainesville, FL, USA; 9 Guts and Glory GNV, Gainesville, FL, USA

**Keywords:** Diversity, storytelling, workforce development

## Abstract

Underrepresentation of Black biomedical researchers demonstrates continued racial inequity and lack of diversity in the field. The *Black Voices in Research* curriculum was designed to provide effective instructional materials that showcase inclusive excellence, facilitate the dialog about diversity and inclusion in biomedical research, enhance critical thinking and reflection, integrate diverse visions and worldviews, and ignite action. Instructional materials consist of short videos and discussion prompts featuring Black biomedical research faculty and professionals. Pilot evaluation of instructional content showed that individual stories promoted information relevance, increased knowledge, and created behavioral intention to promote diversity and inclusive excellence in biomedical research.

## Rationale for Novel Application of Tool/Method or Curricular Approach

Biomedical professionals who identify as Black and understand issues relevant to the Black community are most qualified to produce culturally sensitive research. Yet, the underrepresentation of Black professionals in health professions [1,2] and biomedical research [3,4] is well-documented and demonstrates the continued racial inequity and lack of diversity in the discipline. The literature has identified barriers to inclusion and retainment of Black professionals in the biomedical research workforce, including negative psychosocial and interpersonal dynamics [5], a lack of belonging, feelings of isolation, and societal stereotyping or ingrained systemic disparities. Cultural, social, and psychological forces play a powerful role in whether, or when, Black individuals strive to pursue a career in science [6,7]. Despite awareness of racial disparities and minority recruitment initiatives, there has been minimal progress in promoting racial equity. Black leadership in biomedical research remains low which limits the capacity of research institutions to facilitate health-related endeavors vital to the diverse US population. One aspect of the solution to this issue is to more deeply understand the stories of Black biomedical professionals and to amplify their voices so that they are heard in classrooms, across research campuses, and ultimately across the country.

Storytelling has been used as an important tool for cultural transmission and knowledge sharing throughout human history. It is considered one of the oldest forms of teaching and has allowed thousands of generations to exchange knowledge and values as well as learn from past experiences [8]. Storytelling allows individuals to make sense of the complex world and construct their own meaning via learning from distal events, others’ stories, and past experiences [8,9]. Previous studies have demonstrated that such effects of storytelling can be applied to students’ learning and engagement outcomes. In the context of teaching, storytelling has been considered a foundational approach that facilitates the personal relevance to the knowledge being taught, provides an easy way of comprehension when the information is transmitted in a narrative manner, and creates a more personal connection between the teacher and learners [8]. When receivers are exposed to an interesting story, they are more likely to be immersed in the story and be motivated to remember and internalize the information [10].

## Unmet Need or Educational Gap

The *Black Voices in Research* curriculum addresses two educational and curricular needs: the need for effective instructional materials to promote inclusive excellence [11,12] and the need to facilitate the reflection and dialog about diversity and inclusion in biomedical research. Today’s educational environment calls for colleges and universities to connect their educational quality and inclusion efforts more fundamentally and comprehensively than ever before. As an approach to pedagogy and policy development, inclusive excellence re-envisions both quality and diversity. Specifically, it reflects a motivation for excellence in higher education to be made more inclusive. Decades of work now bring diversity into recruiting, admissions, and hiring, into the main curriculum and supplemental curricular materials, and into administrative structures and practices.

Diversity and inclusion efforts move beyond numbers of students or numbers of programs as end goals, but all too often, information disseminated on racism and racial disparities is presented in the form of statistics, theories, and historical complexities associated with race. Conversely, growing evidence shows that knowledge sharing through personal narratives is more meaningful, memorable, and easier to understand. A powerful story serves as a vehicle for critical conversations and self-reflection. As such, introducing storytelling into educational settings can engage audiences in critical conversations, self-reflection, and bring expanded awareness of the barriers and challenges facing Black professionals in the biomedical field. As an intervention, storytelling is a novel approach to normalizing conversations about behaviors and attitudes towards racism. Curricula promoting and amplifying Black voices thus provide an avenue to foster a collaboration built on trust. It also allows students to develop key insights about the contributing factors shaping identities of Black biomedical researchers, pathways of minority participation and entry into biomedical research, and Black experiences within the biomedical research profession.

## Target Audience

The content of the *Black Voices in Research* curriculum and its narrative storytelling approach can be used as educational material for undergraduate students in STEMM-related fields. It can also be utilized as workforce development and near-peer mentoring materials for research professionals. This curricular approach is an adaptable educational tool that can be used with a wide range of audiences including faculty, professionals, professional, graduate, and undergraduate students, and the general research community. In fact, the inclusion of professionals, researchers, and general audience in the evaluation of the pilot content highlighted the broad relevance of the instructional materials.

## Description of the Educational Method or Curricular Program

The *Black Voices in Research* curriculum was developed within the entertainment–education (E–E) theoretical framework. Traditional definition of E–E refers to entertainment programming that is designed to exert some known and prosocial effect on viewers by embedding prosocial messages into entertainment media content [13]. It has become a popular strategy used in health and education areas to positively influence viewers’ awareness, knowledge, attitudes, or behaviors [13]. When creating the message, E–E does not necessarily come along with a persuasive intent. Rather, it incidentally promotes behavioral change [13]. Considering its narrative nature, E–E messages foster viewers’ involvement with the storyline. Among the different labels describing involvement processes such as absorption, transportation, engagement, and immersion [13], transportation is the most commonly used term that expresses the main idea that viewers are fully involved with the story. Narrative transportation refers to a mental journey that the audience feels as they enter the world being evoked by the narrative. As all mental systems focus on the story plots and characters, transportation occurs when those engaged are reading, watching, or listening to a narrative [14]. The transportation state implies that viewers’ mental systems are fully immersed in the story plots evoked by the narrative, which also indicates E–E is different from story plots that are overly persuasive or purely fact-based [10,13]. Furthermore, previous studies have shown that, because of their resistance-reducing potential, E–E messages may exert a more potent effect on influencing attitudes and behaviors than traditional persuasive messages [13,15].

The curriculum focuses on four learning objectives: 1) Showcase the power of storytelling, particularly for amplifying Black voices; 2) Identify narratives of struggle and thriving shared by Black research professionals; 3) Expand awareness and consciousness of diversity, equity, and inclusive excellence; 4) Apply practical strategies and tools to recognize and promote the stories of race, equity, diversity, and inclusive excellence in different situations. Instructional materials consist of five short videos (5–8 minutes each) and discussion prompts that aim to promote critical thinking, reflection, integration of visions and worldviews, and action to promote diversity and inclusive excellence in biomedical research [15]. The videos were professionally produced by a local production company, Guts and Glory. Storytellers received professional creative training and coaching on using narratives in talking about their professional paths and experiences. The videos feature Black research faculty and professionals engaged in biomedical research, including four Black women and one Black man. The questions presented in discussions are informed by evidence-based dialog methods such as most significant change technique, future search conference, and appreciative inquiry [15]. The curriculum is publicly available for free through the institutional online Canvas learning platform (https://reg.distance.ufl.edu/reg/Activity/Details/e1bf6cb91dad41459f0eb09803813e66).

## Methods of Evaluation

This project was registered in the institutional quality improvement registry. The videos were released on January 14, 2021, and they were promoted as an online event through announcements, registration, and event countdowns. The videos were live streamed through a link sent to the registered attendees. We employed an online survey to evaluate the pilot video content. After watching the videos, participants registered for the online event were sent a link to a 20-item survey securely hosted on the institutional Qualtrics account, and 18% of respondents completed the survey.

The Kirkpatrick model of training evaluation was used as the evaluation framework. The model includes evaluation across four levels: trainee reaction, learning, behavior, and long-term results and outcomes. Level 1, trainee reaction, focuses on the assessment of satisfaction with the training, active engagement with the training content, and relevance of the content to the practical experience beyond the training environment as perceived by trainees. Level 2, learning, focuses on the assessment of knowledge, skills, and attitudes related to the training topics as well as confidence and commitment to the application of knowledge to practice. Level 3, behavior, focuses on the actual application of knowledge to practice and determines the processes and systems reinforcing and encouraging new critical behaviors. Finally, level 4, results and outcomes, measures the degree to which targeted outcomes occur as a result of the training. Effects of the training programs are likely to be realized over a period of months or years. However, short-term observations and measurements can indicate whether critical behaviors are on track to create a positive impact on desired results.

Survey questions included both close- and open-ended to increase the depth of data. The survey questions first asked about participant background (e.g., demographics, home institution). Participants were then asked to comment on their overall satisfaction with the video content, the relevance of each talk, and the length of the videos (i.e., Level 1 training results). Finally, participants were invited to comment on what information resonated with them the most and share their attitudes toward diversity in research (i.e., Level 2).

Close-ended survey questions were analyzed using descriptive statistical approaches. Open-ended textual responses were analyzed using directed qualitative content analysis. For the latter analysis, we developed a codebook with category codes based on the levels of the Kirkpatrick model of training evaluation (see Table 1) [16]. Each response was assigned one category code based on the highest level of training evaluation addressed in the comment. The first author (YLS) coded the data and engaged in discussion with the rest of the authors to ensure the transparency of the analytic process and outcomes.

**Table 1.** Qualitative content analysis codebook based on the levels of Kirkpatrick Model of evaluation

Level	Category	Definition
0	No feedback	No feedback provided
1	Satisfaction	Respondent provides feedback about the technical production and presentation of the event
1	Engagement	Respondent comments about the engagement with and relevance of the topic and content of the event
2	Learning need	Respondent evaluates the knowledge gained from attending the event and comments about the need for additional content
2	Knowledge-skills-attitudes	Respondent comments about the knowledge, skills, and/or attitudes gained through the event
3	Application	Respondent comments about the application of knowledge gained through the event and specific behaviors that resulted from attending the event
3	Dissemination	Respondent acts by disseminating the message thus creating ripple effects and engaging their personal networks with the content
3	Planning	Respondent comments about plans for future actions

## Initial Evidence of Impact on Learning

Survey responses were collected from 90 participants who came from 18 educational and health-related organizations. Participants were predominantly female (*N* = 79; 88%). Racially and ethnically, participants identified as Non-Hispanic White (*N* = 57; 64%), Black (*N* = 21; 23%), Hispanic or Latino (*N* = 7; 7%), Asian (*N* = 2; 2%), and Black Asian (*N* = 1; 1%). The age groups represented included individuals of 18–34 years(*N* = 26; 29%), 35–50 (*N* = 29; 32%), 51–69 (*N* = 29; 32%), and 70 and above (*N* = 6; 7%). The majority of participants were satisfied or highly satisfied (*N* = 84; 93%) and likely to recommend content to others (*N* = 86; 96%). Individual stories were also rated as relevant or highly relevant at a rate of 90% and over.

Qualitative, open-ended feedback was provided by 88 participants. The comments were coded and recoded by the first author to assess intracoder agreement and promote reflexivity [17]. Open-ended feedback addressed Levels 1–3 of the Kirkpatrick model (Table 2 presents counts and representative quotes for each level). For Level 1, participants commented about their *engagement* and *satisfaction* in reaction to the content (i.e., the audience member engaged with and commented on the content or delivery of the content, but did not comment on the new knowledge, skills, or attitudes gained). Specifically, 22 respondents commented about their *engagement* with the content of the event (e.g., “excellent stories”), and another 20 respondents commented about their *satisfaction* with the technical side of the event (e.g., “I thought the staging was dark, but I suppose that was for a purpose.”). Level 2 comments focused on the training results, including new knowledge, skills, or attitudes (*KSA*) or additional *learning needs*. Twelve respondents commented on the *KSAs* gained. They particularly shared about their newly gained commitment and inspiration to address existing discrimination as well as their confidence in engaging in dialogs related to diversity and inclusion in biomedical research. Also related to Level 2 training outcomes were comments about the *learning needs* for additional content and dialog facilitation. Specifically, respondents sought to hear more stories, including from speakers of other minority groups (e.g., LGBTQIA) and other professional backgrounds. Respondents also asked for a facilitated question-and-answer session. Higher levels of evaluation were not expected for this one-time, brief educational intervention. However, a small number of comments were attributed to Level 3 training result, namely the behavior or behavioral intention as the result of the training. Three respondents spoke about the *application* of knowledge, *dissemination* of content, and the *planning* of future actions. Respondents were surveyed immediately after watching the videos and would not have had a chance to act (Level 4) and report behavioral changes. Finally, 11 respondents provided comments with no substantive feedback.

**Table 2.** Categories and counts of open-ended comments based on the Kirkpatrick Model of training results

Level	Category	Count	Representative quote
1	Engagement	22	“Powerful stories!!!!”; “I loved each person’s story and sharing their experiences!”
1	Satisfaction	20	“I thought this event was very well done. It might be interesting to have some imagery along with the stories.”; “I like the programming concept. I thought the staging was dark, but I suppose that was for a purpose.”
2	Knowledge-skills-attitudes	12	“I liked hearing from Black voices. Reading discrimination in third person has never made me as aware of discrimination as hearing this presentation.”; “Reading discrimination in third person has never made me as aware of discrimination as hearing this presentation.”; “Inspiring event! I hope it not only continues to raise awareness but also that more representation of minorities in research and departments […] can occur.”
2	Learning need	18	“I would have loved seeing the speakers together at the end to discuss intersections of identity and common experiences.”
3	Application	3	“Excellent presentation. Thank you for capturing the stories and sharing. We have used this project to help kick off our discussions at the […] CTSI”
3	Dissemination	1	“I loved every minute of it. I shared it on twitter and with my friends/family.”
3	Planning	1	“I thoroughly enjoyed learning more about the skills and experiences of my co-workers. Listening to the acts of discrimination they have experienced wasn't easy, but I hope that hearing their stories will help prevent me from perpetrating similar asks and encourage me to speak of if I am witness to others experiencing similar treatment.”
0	No feedback	11	“None at this time”; “No suggestions”

Evaluation of the long-term effects of this curriculum is an area of future investigation for the project team. Specifically, we plan to conduct evaluation in more controlled settings (e.g., labs and classrooms) and measure immediate (i.e., reaction, attitude change, and behavioral intention), intermediate (i.e., behavior change and actions taken), and longer-term effects measured by academic degree choices and retention rates. For research professionals, long-term impact metrics can include engagement in professional activities (e.g., national organizations, peer mentoring) and biomedical career tenure.

## Discussion

This project has gained wide attention and momentum. At the time of the writing of this paper, the *Black Voices* videos feature nine speakers, and a new event is in the planning. The videos have been publicly shared on YouTube and viewed almost 5500 times. The initial evaluation of the audience reaction to the content shows that the sharing of stories and Black voices in research creates an opportunity for learning, reflection, and dialog around diversity and inclusion in biomedical and translational research. Comments from the audience also suggest that there is a need to supplement the instructional content of *Black Voices* with structured discussions, which could be organized and delivered as workshops or classroom modules. The short-term outcome we pursued was the increased awareness of the multifaceted careers in biomedical research for Black professionals and other minorities. The long-term impact of this curriculum is to facilitate amplification and dissemination of Black voices and to promote diversity and inclusive excellence in biomedical research. The indicators of such potential long-term impacts can include attitudes toward research careers, degree choices made by early-stage trainees, and retention of minority trainees within STEMM disciplines.

In conclusion, past research clearly demonstrates that the way one tells their story can serve as a vehicle for critical conversations, self-reflection, and well-being [18]. Such programming benefits not only our current and future Black biomedical professionals, but also the audience who are privileged to hear and grow from such story exchange. Stories play an important role in structuring our experience and cultural learning, as we learn from stories and interact with the world without leaving current situations. *Black Voices in Research* provides behavioral exemplars for Black students who will have more access to visible leaders and role models in their fields of interest and may feel more confident in pursuing biomedical science.
